# Insights Into the Microbial Degradation and Biochemical Mechanisms of Neonicotinoids

**DOI:** 10.3389/fmicb.2020.00868

**Published:** 2020-05-19

**Authors:** Shimei Pang, Ziqiu Lin, Wenping Zhang, Sandhya Mishra, Pankaj Bhatt, Shaohua Chen

**Affiliations:** ^1^State Key Laboratory for Conservation and Utilization of Subtropical Agro-bioresources, Guangdong Province Key Laboratory of Microbial Signals and Disease Control, Integrative Microbiology Research Centre, South China Agricultural University, Guangzhou, China; ^2^Guangdong Laboratory for Lingnan Modern Agriculture, Guangzhou, China

**Keywords:** neonicotinoids, toxicity, microbial degradation, metabolic pathways, bioremediation, molecular mechanisms

## Abstract

Neonicotinoids are derivatives of synthetic nicotinoids with better insecticidal capabilities, including imidacloprid, nitenpyram, acetamiprid, thiacloprid, thiamethoxam, clothianidin, and dinotefuran. These are mainly used to control harmful insects and pests to protect crops. Their main targets are nicotinic acetylcholine receptors. In the past two decades, the environmental residues of neonicotinoids have enormously increased due to large-scale applications. More and more neonicotinoids remain in the environment and pose severe toxicity to humans and animals. An increase in toxicological and hazardous pollution due to the introduction of neonicotinoids into the environment causes problems; thus, the systematic remediation of neonicotinoids is essential and in demand. Various technologies have been developed to remove insecticidal residues from soil and water environments. Compared with non-bioremediation methods, bioremediation is a cost-effective and eco-friendly approach for the treatment of pesticide-polluted environments. Certain neonicotinoid-degrading microorganisms, including *Bacillus*, *Mycobacterium*, *Pseudoxanthomonas*, *Rhizobium*, *Rhodococcus*, *Actinomycetes*, and *Stenotrophomonas*, have been isolated and characterized. These microbes can degrade neonicotinoids under laboratory and field conditions. The microbial degradation pathways of neonicotinoids and the fate of several metabolites have been investigated in the literature. In addition, the neonicotinoid-degrading enzymes and the correlated genes in organisms have been explored. However, few reviews have focused on the neonicotinoid-degrading microorganisms along with metabolic pathways and degradation mechanisms. Therefore, this review aimed to summarize the microbial degradation and biochemical mechanisms of neonicotinoids. The potentials of neonicotinoid-degrading microbes for the bioremediation of contaminated sites were also discussed.

## Introduction

The increasing global demand for food and productive crops has facilitated tremendous growth of the pesticide sector. Neonicotinoid insecticides have been widely used for the protection of crops from a variety of insects and pests, to enhance production (Simon-Delso et al., 2015). These crops include rice, wheat, maize, soybean, cotton, sugar beet, apple, potato, etc. Neonicotinoid insecticides are developed and synthesized based on nicotine structure research with better insecticidal capabilities (Humann-Guilleminot et al., 2019). Neonicotinoids are a kind of neuroactive insecticide. Their insecticidal mechanism involves the action of nicotine acetyl bile on the postsynaptic membrane of insect nicotinic acetylcholine receptors (nAChRs) whereas the surrounding nerves induce excitation leading to paralysis and death (Zhang et al., 2018). There is no cross-resistance to the traditional long-acting insecticide classes due to the user mode of action (MoA). Therefore, neonicotinoids replace those of organochlorine, organophosphorus, chlorinated hydrocarbons, carbamate, pyrethroid insecticides, and several other chemical categories (Jeschke and Nauen, 2008). In the mid-1980s, Bayer contributed to imidacloprid (1-[(6-chloro-3-pyridinyl)-methyl]-*N*–nitro-2-imidazolidinimine), which was the first neonicotinoid insecticide, and this captured one of the highest shares in the global pesticide market (Peter et al., 2010). Worldwide, neonicotinoids accounted for approximately one-quarter of the pesticide market, and their annual production was about 600,000 tons (Simon-Delso et al., 2015). The broad-spectrum characteristics and high insecticidal activity of imidacloprid led to the development of a series of nicotinic insecticides, including thiamethoxam, clothianidin, dinotefuran, acetamiprid, thiacloprid, and nitenpyram.

Neonicotinoids include three main compounds:

(1) Chloropyridinyl compounds (imidacloprid, nitenpyram, acetamiprid, and thiacloprid),
(2) Chlorothiazolyl compounds (thiamethoxam and clothianidin), and
(3) Tetrahydrofuran compounds (dinotefuran) (Table 1).

Neonicotinoids are commonly used for seed treatment, soil wetting, and foliage sprays to protect crop seedlings from leaf-eating insects (Hussain et al., 2016). Neonicotinoids are water-soluble at concentrations of 184–590.0 mg⋅L^–1^ at 20°C and pH 7. Therefore, neonicotinoids are absorbed and circulated throughout the plant system to protect against insects (Ellis et al., 2017). Neonicotinoids bind and activate the postsynaptic nAChR of insects, which causes muscle tremors and cell fatigue. The effect of neonicotinoids is significantly stronger on invertebrates than vertebrates as vertebrates have more nAChRs (Taillebois et al., 2018; Zaworra et al., 2018). Neonicotinoids are efficient insecticides with low toxicity but their long-term usage has generated extensive environmental problems (Mariusz et al., 2013). These compounds not only affect the growth of plants and animals but also induce changes in the gene expressions (Mikolić and Karačonji, 2018).

**TABLE 1:** Neonicotinoid compounds and their chemical structures and molecular formulae.

Compound name	Molecular mass (g⋅mol^–1^)	Molecular formula	Chemical structure
Imidacloprid	255.67	C_9_H_10_ClN_5_O_2_	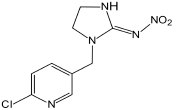
Acetamiprid	222.68	C_10_H_11_ClN_4_	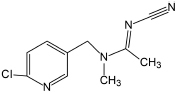
Dinotefuran	202.21	C_7_H_14_N_4_O_3_	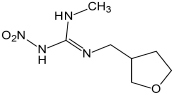
Clothianidin	249.68	C_6_H_8_ClN_5_O_2_S	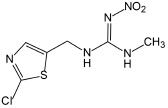
Thiacloprid	252.72	C_10_H_9_ClN_4_S	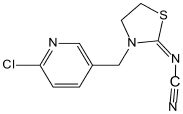
Thiamethoxam	291.71	C_8_H_10_ClN_5_O_3_S	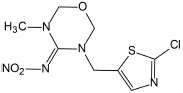
Nitenpyram	270.72	C_11_H_15_ClN_4_O_2_	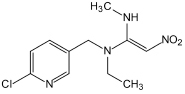

Decades of extensive use of neonicotinoids have resulted in a universal phenomenon in the environment, posing a dangerous threat to humans and ecosystems (Casida, 2017; Humann-Guilleminot et al., 2019). These insecticides are considered a key factor in diminishing pollinating insects, especially honeybees (Friedli et al., 2020). Neonicotinoids can be adhered easily to the surface of plants, animals, and human beings, and do not degrade easily into the environment (Hladik et al., 2018). Due to the toxic and hazardous effects to bees, the outdoor use of neonicotinoids is banned in European Union member states.

Usually, both biotic and abiotic factors, including chemicals, sunlight, and microbial agents, promote the degradation of native and foreign organisms in the soil (Chen et al., 2015; Birolli et al., 2018; Yang et al., 2018; Zhan et al., 2018a; Bhatt et al., 2020c; Zhang et al., 2020). Microbial degradation is often used to transform synthetic chemicals into inorganic products (Chen et al., 2011, 2013; Arora et al., 2017; Cycoñ et al., 2017; Huang et al., 2019; Feng et al., 2020a). Uncontrollable photocatalytic conditions are the major disadvantage of non-biological degradation and thus microbial degradation emerges as a better alternative (Zhang et al., 2018). It is usually insufficient for analytical procedures to report the concentrations as most countries generally lack the environmental monitoring data systematically for neonicotinoids.

Individual neonicotinoid concentrations from the water monitoring literature demonstrated average surface water concentrations of 0.13 μg⋅L^–1^ (*n* = 19 studies) and a peak surface water concentration of 0.63 μg⋅L^–1^ (*n* = 27 studies) (Christy et al., 2015). The fate of neonicotinoid insecticides in the soil environment is greatly decided by the microbial metabolisms. However, bacteria with a complete set of genes required for complete mineralization have not been found yet. Soil microbial degradation processes of imidacloprid, acetamiprid, and thiacloprid have been understood; however, little has been reported regarding the microbial degradation of clothianidin, dinotefuran, thiamethoxam, and nitenpyram (Mulligan et al., 2016).

The microbial degradation of neonicotinoids is considered to be the most efficient and environmentally friendly *in situ* repair pathway (Hamada et al., 2019). The use of potential and degradative microorganisms, which can grow and survive under high-stress concentrations of insecticides, offers a possible opportunity for the remediation of toxic pollutants and hazardous wastes from contaminated environments (Chen et al., 2014; Rana et al., 2015; Xiao et al., 2015; Zhan et al., 2018b; Bhatt et al., 2020a).

A variety of neonicotinoid-degrading microorganisms have been isolated and identified. These microbes include *Bacillus*, *Mycobacterium*, *Pseudoxanthomonas*, *Rhizobium*, *Rhodococcus*, *Actinomycetes*, and *Stenotrophomonas* (Dankyi et al., 2018; Zhang et al., 2018; Hamada et al., 2019). The bioremediation potential of neonicotinoid-contaminated water/soil environments using several different microbes has been investigated. However, the detailed knowledge about specific degradative enzymes and genes still needs to be explored. To date, only a few studies have focused on the enzymatic and genetic basis of neonicotinoid-degrading microorganisms to evolve better pathways for sustainable degradation. Hence, in this review article, the microbial degradation pathways of neonicotinoids were summarized, and the molecular biology and genetic pattern of neonicotinoid-degrading microbes were also discussed.

## Toxicity of Neonicotinoids

At present, neonicotinoids have developed into one of the most widely used insecticides all over the world and have attracted a great deal of attention for their cumulative toxic effects and drug resistance (Bartlett et al., 2019; Li et al., 2019; Wang et al., 2019a,b,c). It is mainly used to control mealworm planthopper, leafhopper, aphid, psylla thrips, leaf beetle, leaf miner, leaf beetle, beetle, termite, red fire ant, cockroach, fly, nematode, and other pests (Calvo-Agudo et al., 2019). Compared to the original pesticide, several neonicotinoid metabolites possess stronger toxicity and durability (Hamada et al., 2019). Only about 10% of the applied insecticide reaches target organisms, and the remaining 90% is distributed in the environment where it can adversely affect non-target organisms and ecosystems (Lin et al., 2020). Therefore, the bioavailability of pesticides in the soil environment is an important factor affecting soil microbial population.

To assess the genetic, structural, and functional biodiversity of the microbial community in neonicotinoid (imidacloprid) treated soils various biotechnological and molecular approaches, including the use of phospholipid fatty acid (PLFA) profiles, denaturing gradient gel electrophoresis (DGGE), and community level physiological profiles (CLPP), have been performed. Among these techniques, PLFA profiles are very useful to give the information about the shifting of microbial community structure and decrease in the total biomass of the microbial community after imidacloprid treatment (Cycoñ and Piotrowska-Seget, 2016). Toxicological studies of neonicotinoids have been conducted on flying organisms, aquatic organisms, terrestrial organisms, and human beings.

This paper summarized the representative studies of the last 3 years (Table 2) and concluded that high levels of neonicotinoid pesticides harmfully affected non-target biological bees during pollen harvesting and flight (Zaworra et al., 2018; Colin et al., 2019). Moreover, long-term exposure to neonicotinoid pesticides led to weight loss, impaired flight, and phototropism. The accumulation of neonicotinoids was demonstrated to severely affect their longevity, pollination, and learning ability, and suppress gene expression (Ellis et al., 2017; Tosi et al., 2017; Tosi and Nieh, 2017; Christen et al., 2018; Manjon et al., 2018; Li et al., 2019). Other non-target organisms may also be seriously affected by these pesticides. The European Siberian carabid *Platynus assimilis* exercised less after a short period of hyperactivity due to the neonicotinoid pesticide (Tooming et al., 2017). The survival and growth of the mayfly were significantly reduced by sub-lethal concentrations of neonicotinoid pesticides (Bartlett et al., 2018).

**TABLE 2:** Toxicological studies of neonicotenoid compounds.

No.	Neonicotinoid	Sample source/study sample	Specific statement	References
1	Thiamethoxam	Honey Bee Queen	Honey bee queen’s body weight, ovary weight, and sperm counts were significantly reduced	Gajger et al., 2017
2	Thiacloprid, acetamiprid, thiamethoxam, clothianidin	Mice and rabbits	Pesticides were toxic to mice and rabbit embryos	Babeíová et al., 2017
3	Imidacloprid and thiacloprid	*Folsomia candida*	Causes toxicity to three generations of *Folsomia candida*	van Gestel et al., 2017
4	Dinotefuran	Earthworms	Lipids, proteins and nucleic acids were oxidized and destroyed by the production of large amounts of ROS	Liu et al., 2017
5	Thiacloprid, thiamethoxam, imidacloprid	H295R cells of human	Inhibition of 16α-hydroxylation of fetal DHEA	Caron-Beaudoin et al., 2017
6	Thiamethoxam	European Siberian carabid *Platynus assimilis*	Insects exercised less after a short period of hyperactivity	Tooming et al., 2017
7	Thiamethoxam	Bumblebee	Leads to premature death and weigh less to survive	Ellis et al., 2017
8	Thiamethoxam	Honey bee	Flight ability and phototropism were seriously affected	Tosi et al., 2017
9	Clothianidin, imidacloprid, thiamethoxam	Honey bee	Gene expression was seriously affected	Christen et al., 2018
10	Imidacloprid	Zebrafish	Both gene expression and protein levels were increased	Selçuk et al., 2018
11	Cycloxaprid	Earthworm	The epidermis, gut and neurochord were damaged, and enzyme activities of catalase and superoxide dismutase were effected	Qi et al., 2018
12	Acetamiprid, clothianidin, imidacloprid, thiacloprid, thiamethoxam	HepG2 and SH-SY5Y cells	Cytotoxicity and DNA damage	Şenyildiz et al., 2018
13	Imidacloprid	Freshwater microcosms	Numbers and abundance of microorganisms species were decreased.	Sumon et al., 2018
14	Thiamethoxam	Mongolian Racerunner	Carcinogenic and hepatic injury risk	Wang et al., 2018
15	Imidacloprid, nitenpyram	*Gobiocypris rarus*	8-OHdG content and AChE activity was increased at 2.0 mg⋅L**^–^**^1^ imidacloprid but AChE activity was decreased at 2.0 mg⋅L**^–^**^1^ nitenpyram	Tian et al., 2018
16	Imidacloprid, nitenpyram, dinotefuran	Chinese rare minnows	Induces genotoxicity and decrease immune system	Hong et al., 2018
17	Thiacloprid	Hs578t cells	Stimulate a change in CYP19 promoter	Caron-Beaudoin et al., 2018
18	Clothianidin	Mice	Results in anxiety-related behavior and can increase some parts of thalamine and hippocampal regions	Hirano et al., 2018
19	Imidacloprid, thiamethoxam, acetamiprid, clothianidin, thiacloprid, dinotefuran	Mayfly	At sub-lethal concentrations, survival and growth of mayfly were significantly reduced	Bartlett et al., 2018
20	Imidacloprid	Fish	Results in DNA damage above 100 μg⋅L**^–^**^1^ Concentration	Iturburu et al., 2018
21	Imidacloprid	Zebrafish, medaka	Sublethal effects in both species but the effects were much stronger in medaka with deformities, lesions and reduced growth being the most prominent impacts	Vignet et al., 2019
22	Dinotefuran, thiamethoxam, imidacloprid, clothianidin	Chinese lizards	Dinotefuran and thiamethoxam directly increased the concentrations of acetylcholine in brain and blood, and clothianidin aggravated neurotoxic effects of thiamethoxam	Wang et al., 2019a
23	Imidacloprid	Honey bee	Learning ability and some gene expression were suppressed	Li et al., 2019
24	Imidacloprid, thiamethoxam, acetamiprid, clothianidin, thiacloprid, dinotefuran	Freshwater amphipod	Neonicotinoids reduced the survival whereas clothianidin and acetamiprid were the most toxic insecticides	Bartlett et al., 2019
25	Thiamethoxam, clothianidin	*Mongolian racerunners*	Thiamethoxam and clothianidin interfered with endocrine system	Wang et al., 2019c
26	Dinotefuran, thiamethoxam, imidacloprid	Farmland lizard	Dinotefuran damaged liver and interfered in GH/IGF pathway whereas imidacloprid severely damaged liver oxidative stress	Wang et al., 2019b
27	Imidacloprid	Honey bee	Disrupt colony function, by effecting the division of labor and reducing foraging efficiency	Colin et al., 2019
28	Nitenpyram	Zebrafish liver	Affect antioxidant enzymes and causes DNA damage	Yan et al., 2015
29	Dinotefuran	Honey bee	Long-term exposure leads to neurotoxic effects	Liu et al., 2019

Neonicotinoids pose a significant toxicological impact on fish and other aquatic microorganisms (van Gestel et al., 2017; Caron-Beaudoin et al., 2018; Vignet et al., 2019). These were also reported to induce genotoxicity and reduce the immune system in aquatic organisms (Hong et al., 2018). The exposure of fish to neonicotinoids led to increased gene expression and protein levels; whereas, AChE activity decreased (Selçuk et al., 2018; Tian et al., 2018). van Gestel et al. (2017) reported that high concentrations of imidacloprid and thiacloprid caused toxicity for up to three generations of *Folsomia candida*. The toxicity of neonicotinoids also significantly reduced the number of microorganisms and the abundance of phytoplankton and zooplanktons (Sumon et al., 2018).

Neonicotinoid pesticides can affect land animals by direct contact or food chain transmission. Mollusks and earthworms are more prone to these pesticides as their body surface comes in direct contact with a large area of soil. The oxidation and destruction of lipids, proteins, and nucleic acids in earthworms in response to large amounts of dinotefuran has been well reported (Liu et al., 2017). Qi et al. (2018) have further explained the damage of the epidermis, gut, and neurochord in earthworms by cycloxaprid along with effected catalase and superoxide dismutase enzymes activities.

A variety of neonicotinoid pesticides are known to produce carcinogenic and neurotoxic effects in lizards by damaging the liver and interfering in the growth hormone/insulin-like growth factor (GH/IGF) pathway (Wang et al., 2018, 2019a,b,c, 2020). Thiamethoxam and clothianidin also interfere with the endocrine system (Wang et al., 2019a, c), can increase the thalamic and hippocampal regions of mice, and pose toxic effects on the embryos of mice and rabbits (Babeíová et al., 2017; Hirano et al., 2018).

Şenyildiz et al. (2018) reported that neonicotinoid insecticides might also induce cytotoxicity and DNA damage in mammalian cells. The exposure of human neuroblastoma (SH-SY5Y) as well as human hepatocellular carcinoma (HepG2) cells to neonicotinoid pesticides brings about damage of the SHSY-5Y cells and alteration of the HepG2 cells (Şenyildiz et al., 2018; Bivehed et al., 2019). The exposure of Hs578t cells to environmental concentrations of neonicotinoid results in the inhibition of I.4 promoter activity and aromatase catalytic activity (Caron-Beaudoin et al., 2018). Neonicotinoid pesticides also cause reproductive toxicity in humans by increasing the estrone and estradiol production and strongly inhibiting estriol production (Caron-Beaudoin et al., 2017; Han et al., 2018).

## Neonicotinoid-Degrading Microorganisms

Due to their characteristics of high applicability, ease of operation, and low cost, microbial degradation is acceptable in practical applications for the degradation of large amounts of neonicotinoids that remain in the environment (Shaikh et al., 2014; Jean-Marc et al., 2019). As one of the potential applications in the degradation of pollutants, cell immobilization (CI) is an effective method characterized by limiting cells to a defined region while maintaining their metabolic, catabolic, and catalytic activity. Immobilized cells can degrade toxic substances more efficiently than free cells (Conde-Avila et al., 2020). Under the condition of conventional bacteriological media and harsh environmental conditions, most bacteria cannot be cultured and enter into a viable but non-culturable (VBNC) state (Ramamurthy et al., 2014; Fida et al., 2017; Su et al., 2018, 2019). A VBNC state could be used to improve the biodegradation of neonicotinoids. There are two categories of bacterial biodegradation: (a) pure bacterial culture biodegradation and (b) microbial co-degradation (Table 3).

**TABLE 3:** Degradation studies of neonicotinoid compounds by isolated microorganisms.

No.	Microorganisms	Type	Isolation source	Mode of degradation	Comment	References
**Neonicotinoids**						
1	*Acinetobacter* sp. TW	Bacterium	Solid tobacco waste	Catabolic ^∗^(C, N)	Degraded acetamiprid and imidacloprid under broad pH and temperature conditions	Wang et al., 2011
2	*Sphingomonas* sp. TY	Bacterium	Solid tobacco waste	Catabolic ^∗^(C, N)	Degraded acetamiprid and imidacloprid under broad pH and temperature conditions	Wang et al., 2011
**Imidacloprid**						
3	*Pseudomonas* sp. 1G	Bacterium	Neonicotinoid exposed golf course soil	Co-metabolic (glucose)	28°C, microaerophilic	Pandey et al., 2009
4	*Bacillus aerophilus*	Bacterium	Sugarcane field soils	Co-metabolic; mixed culture	Soil slurry	Akoijam and Singh, 2015
*5*	*Bacillus alkalinitrilicus*	Bacterium	Sugarcane fields	Catabolic ^∗^(C, N)	Used mixed culture of native soil	Sharma et al., 2014
6	*Bradyrhizobiaceae* strain SG-6C	Bacterium	Soil	Catabolic ^∗^(C)	Degraded 6-chloronicotinic acid	Shettigar et al., 2012
7	*Ochrobactrum* BCL-1	Bacterium	Tea rhizosphere soil	Catabolic ^∗^(C)	Degraded 50% imidacloprid (50 g⋅L**^–^**^1^) in the culture within 26 h and approximately 70% within 48 h	Hu, 2013
*8*	*Klebsiella pneumonia* BCH-1	Bacterium	Pesticide contaminated agricultural field	Co-metabolic	pH 7, 30°C, static condition	Phugare et al., 2013
9	*Leifsonia* strain PC- 21	Bacterium	Monona soil	Co-metabolic (glucose, succinate)	Degrade 37% - 58% of Imidacloprid in the full strength TSB	Anhalt et al., 2007
*10*	*Mycobacterium* sp. strain MK6	Bacterium	Agricultural soil	Catabolic ^∗^(N)	Liquid minimal medium	Kandil et al., 2015
*11*	*Pseudoxanthomonas indica* CGMCC 6648	Bacterium	Rhizospheric soils	Co-metabolic (glucose)	Liquid minimal medium	Ma et al., 2014
*12*	*Rhizobium* sp.	Bacterium	Vegetable farming areas	Catabolic ^∗^(C)	Liquid minimal medium	Sabourmoghaddam et al., 2015
*13*	*Pseudomonas* sp. RPT 52	Bacterium	Pesticide contaminate agricultural field	Catabolic ^∗^(C)	Degradation rate of endosulfan > coragen > imidacloprid	Gupta et al., 2016; Manasi et al., 2016
*14*	*Aspergillus terreus* YESM3	Fungus	Agricultural wastewater	Catabolic ^∗^(C)	28°C, pH 4	Mohammed and Badawy, 2017
*15*	*Stenotrophomonas maltophilia* CGMCC 1.1788	Bacterium	China General Microbiological Culture Collection Center	Co-metabolic	30°C, pH 7.2	Dai et al., 2006
*16*	*Hymenobacter latericoloratus* CGMCC 16346	An obligate oligotrophic bacterium	Water environment	Co-metabolic	64.4% of imidacloprid was degraded in 6 days	Guo et al., 2020
**Acetamiprid**						
*17*	*Ochrobactrum* sp. D-12	Bacterium	Wastewater treatment pool	Catabolic	30−45°C, pH 5−10, identification of dechlorinated metabolite	Yang et al., 2013
18	*Phanerochaete sordida* YK-624	Fungus	Rotted wood	*N*-demethylated	Biotransformation of acetamiprid by a white-rot fungus	Wang et al., 2012
*19*	*Rhodotorula mucilaginosa* Strain IM-2	Yeast	Soil	No data	Biodegraded acetamiprid and thiacloprid in soils	Dai et al., 2010
20	*Pigmentiphaga* sp. AAP-1	Bacterium	Pesticide contaminated factory soil	Catabolic	30°C, resting cells, pH 7	Wang et al., 2013a
21	*Pigmentiphaga* sp. D-2	Bacterium	Wastewater treatment pool	Catabolic ^∗^(C)	30−45°C, pH 5−10	Yang et al., 2013
22	*Ensifer meliloti* CGMCC7333	Bacterium	Rhizosphere soils	Catabolic ^∗^(N) N-aminoamide IM-1-2	Nitrogen- fixing	Zhou et al., 2014
23	*Pseudoxanthomonas* sp. AAP-7	Bacterium	Polluted soil	Co-metabolic	30°C, resting cells, pH 7	Wang et al., 2013b
24	*Rhodococcus* sp. BCH-2	Bacterium	Pesticide contaminated soil	Co-metabolic 6-chloronicotinic acid	35°C, pH 7, static	Phugare and Jadhav, 2013
25	*Pseudomonos* sp. FH2	Bacterium	Agriculture field soil	Catabolic	Spiked imidacloprid (50 μg⋅mL^–1^)	Yao and Min, 2006
26	*Stenotrophomonas* sp. THZ-XP	Bacterium	Sludge from an Acetamiprid producing factory	Co-metabolic	30°C, pH 7	Tang et al., 2012
27	*Fusarium* sp. strain CS-3	Fungus	Soil from pesticide factory	Co-metabolic (glucose, peptone)	25−30°C, pH 5.0−7.0	Shi et al., 2018
28	*Stenotrophomonas maltophilia* CGMCC 1.1788	Bacterium	China General Microbiological Culture Collection Center	Co-metabolic	30°C, pH 7.2	Chen et al., 2008
29	*Actinomycetes Streptomyces canus* CGMCC 13662	Actinomycete	Soil	Co-metabolic	pH 7, 30°C	Guo et al., 2019
30	*Variovorax boronicumulans* CGMCC 4969	Bacterium	China General Microbiological Culture Collection Center	Co-metabolic	pH 7, 40°C	Sun S.-L. et al., 2018
**Thiacloprid**						
31	*Ensifer meliloti* CGMCC7333	Bacterium	Rhizosphere soils	Catabolic ^∗^(N)	30°C	Ge et al., 2014
32	*Stenotrophomonas maltophilia* CGMCC1.178	Bacterium	China General Microbiological Culture Collection Center	Co-metabolic	30°C, pH 7.2 (resting cells)	Zhao et al., 2009
33	*Variovorax boronicumulans* J1	Bacterium	Agricultural soils	Co-metabolic (resting cells)	30°C, pH 7.2	Zhang et al., 2012
*34*	*Microvirga flocculans* CGMCC 1.16731	Bacterium	Thiacloprid-contaminated soil	Co-metabolic	Transformed 90.5% of 0.63 mmol⋅L^–1^ thiacloprid in 30 h	Zhao et al., 2019
**Thiamethoxam**						
*35*	*Ensifer adhaerens* TMX-23	Bacterium	Agricultural soils	Catabolic ^∗^(C, N)	30°C	Zhou et al., 2013
*36*	*Pseudomonas* sp. 1G	Bacterium	Neonicotinoid exposed golf course soil	Co-metabolic	28°C, microaerophilic	Pandey et al., 2009
*37*	*Bacillus aeromonas* IMBL 4.1	Bacterium	Agricultural soils	Catabolic	pH 6.0−6.5 37°C	Rana et al., 2015
*38*	*Pseudomonas putida* IMBL 5.2	Bacterium	Agricultural soils	Catabolic	pH 6.0−6.5 37°C	Rana et al., 2015
*39*	*Acinetobacter* sp. TW	Bacterium	Agricultural soils	Catabolic	pH 6.0−6.5 37°C	Rana et al., 2015
*40*	*Sphingomonas* sp. TY	Bacterium	Agricultural soils	Catabolic	pH 6.0−6.5 37°C	Rana et al., 2015
**Clothianidin**						
41	*Pseudomonas stutzeri* smk	Bacterium	Agricultural soil	Catabolic ^∗^(C)	pH 7 and 30°C	Parte and Kharat, 2019
42	*Phanerochaete sordida*	Fungus	Rotted wood	N-demethylated	37% clothianidin was degraded at 30°C in 20 days	Mori et al., 2017
**Nitenpyram**						
43	*Phanerochaete sordida* YK-624	Fungus	Rotted wood	Catabolic	100% degradation under ligninolytic conditions	Wang J. et al., 2019
**Dinotefuran**						
44	*Phanerochaete sordida* YK-624	Fungus	Rotted wood	Catabolic	31% degradation under ligninolytic conditions	Wang J. et al., 2019

* C, carbon source; N, nitrogen source.

Pure bacterial biodegradation uses pesticides as the only source of nitrogen or carbon needed for growth, whereas co-metabolic biodegradation requires more nutrition sources, in addition to pesticides. According to the chemical structure of pesticides and the specific environmental conditions to reduce the decomposition activity of microorganisms, neonatal metabolites may vary greatly (Hussain et al., 2016). A clear understanding of the degradation kinetics is important for further enhancement of the degradation (Wang et al., 2013b). Rhizobacterial inoculants have been mainly used for controlling plants and biology. Recently, it was reported that the inoculation of plants with specific plant-growth-promoting rhizobacteria (PGPR) strains enhanced the absorption of ingredients (N, P, K, Fe, Zn, and Mg), heavy metals (Cd, Ni, and Pb), and pesticides. However, research on the relationship between plant growth promotion by PGPR inoculants and enhanced absorption is being further studied (Myresiotis et al., 2015).

### Imidacloprid

Imidacloprid, (1-[(6-chloro-3-pyridinyl)-methyl]-*N*-nitro-2-imidazolidinimine), is a kind of super-efficient neonicotinoid with colorless crystals and a faint smell. It is a broad-spectrum, widely used, and has comparatively lower toxicity and residues. Imidacloprid is relatively safe for humans and animals, and it has not been reported with pest resistance. It is mainly used against sucking pests of rice, wheat, and cotton crops including aphids, leafhoppers, thrips, whitefly, potato beetle, and straw fly.

Imidacloprid blocks the central nervous conduction in insects and induces paralysis leading to death. The decrease of the bioavailability of microbial-degradable pesticides led to the extension of the half-life of imidacloprid and its metabolites in soil (Anhalt et al., 2007). The report on microbial degradation of imidacloprid by *Leifsonia* sp. strain PC-21 was isolated from contaminated sites by an enrichment cultivation technique that degraded 70% of imidacloprid within 14 days and formed desnitro and urea metabolites (Anhalt et al., 2007). *Leifsonia* strain PC-21 was also reported to degrade about half of 25 mg⋅L^–1^ imidacloprid in tryptic soy broth containing 1 g⋅L^–1^ succinate and D-glucose at 27°C in 3 weeks (Phugare et al., 2013).

6-Chloronicotinic acid (6-CNA), olefinic cyclic nitroguanidine, cyclic urea, cyclic guanidine, nitroso, and nitro derivatives are major imidacloprid metabolites, which have been detected in soil and water samples (Kandil et al., 2015; Wang et al., 2016; Seifrtova et al., 2017). In the same way, *Pseudomonas* sp. 1G can also convert imidacloprid into denitrification products and urea metabolites (Pandey et al., 2009). *Stenotrophomonas maltophilia* induces the hydroxylation of imidacloprid to generate 5-hydroxyl imidacloprid, which has a stronger insecticidal activity than the parent compound (Dai et al., 2006).

The Bradyrhizobiaceae strain SG-6C can utilize 6-CNA as the sole carbon source and a 1% (*v*/*v*) seed culture of this strain completely degraded 20 mg⋅L^–1^ of 6-CNA (0.1 mM) within 152 h (Shettigar et al., 2012). Phugare et al. (2013) reported that the *Klebsiella pneumoniae* strain BCH1 degraded 78% imidacloprid within 7 days at 30°C. *Bacillus*, *Bacillus brevis*, *Pseudomonas* sp. F1, *Bacillus subtilis* and *Rhizobia* degraded 25%–45% initial imidacloprid (25 g⋅L^–1^) in carbon limited minimal salt medium (MSM) within 25 days (Sabourmoghaddam et al., 2015).

In addition to pure cultures, mixed cultures of native soil bacteria (*Bacillus aerophilus* and *Bacillus alkalinitrilicus*) have also been reported for the remediation of imidacloprid-contaminated soils (Sharma et al., 2014; Akoijam and Singh, 2015). As a sole source of carbon and energy, 46.5% of 0.5 mM imidacloprid in a minimal medium was degraded by *Pseudomonas* sp. RPT 52 at more than 40 h. Rhizospheric microorganisms are potent degraders of environmental contaminants due to their unique resistance properties. *Pseudoxanthomonas indica* isolated from rhizospheric soil was reported with the fastest imidacloprid biodegradation rate (2.17 μg⋅mL^–1^⋅h^–1^) in liquid culture and soil slurry as compared to other bacterial strains (Ma et al., 2014).

According to the latest research, Guo et al. (2020) isolated an Oligotrophic bacterium, *Hymenobacter latericoloratus* CGMCC 16346, from a water environment. This bacterium can survive for a long time on 1/10,000 diluted nutrient medium and degrade imidacloprid in apotrophic surface water. In addition to bacteria, fungal cultures are also effective tools in the bioremediation of imidacloprid. The effective bioremediation of imidacloprid from contaminated water and environmental samples with *Aspergillus terreus* YESM3 was reported (Mohammed and Badawy, 2017).

Biodegradation is one of the best techniques that works well under a laboratory environment (Liu et al., 2015; Bhatt et al., 2020b; Lin et al., 2020). Sometimes, the incompatibility of microorganisms with the environment under field conditions reduces their efficiency. Indigenous microorganisms of an environment can easily replace applied cultures because of the difference between the *in situ* and *ex situ* environments of the plants, the soil properties, and the micro-ecological conditions (Chen et al., 2012; Bhatt et al., 2020c; Huang et al., 2020; Zhan et al., 2020). Therefore, it is very essential to study the compatibility of isolated strains.

### Acetamiprid

Acetamiprid, *N*-(*N*-cyano-ethyleneimine)-*N*-methyl-2-chloropyridine-5-methylamine, is a new kind of broad-spectrum neonicotinoid insecticide with acaricidal activity. It is a systematic insecticide of soil, branches, and leaves, and is widely used to control lepidopteron pests of rice, and planthoppers of vegetables, fruits, and tea. Hussain et al. (2016) summarized the microbial degradation process of neonicotinoids in soil and water environments through bacterial communities. The important environmental factors for the microbial degradation of xenobiotic compounds are temperature and pH (Phugare and Jadhav, 2013).

Several optimization studies of the physicochemical parameters showed that a temperature of 35°C and a pH of 7.0 was optimal for acetamiprid biodegradation. Moreover, the degradation of acetamiprid was studied under different temperatures and pH conditions by strain CS-3. The optimum pH-value for acetamiprid degradation ranged from 5.0 to 7.0, and the degradation value decreased when the pH-value was above 8.0 or below 4.0. Strain CS-3 efficiently degraded acetamiprid between 25 to 30°C, whereas the degradation rate was reduced to half at 20°C and 42°C (Shi et al., 2018).

Acetamiprid rapidly degraded through aerobic soil metabolism with a half-life of 1–8.2 days during various soil studies in the United States and Europe, whereas it took 16–17 days under the field conditions of India (Gupta and Gajbhiye, 2007). *Pseudomonos* sp. FH2, isolated by Yao and Min (2006), effectively degraded 96.7% acetamiprid in 14 days at pH 7 and 30°C temperature. The transformation rates of acetamiprid by *Stenotrophomonas* sp. THZ-XP (Tang et al., 2012) and *Pigmentiphaga* sp. AAP-1 (Wang et al., 2013a) were faster than other strains.

*Stenotrophomonas maltophilia* CGMCC 11788 had the highest imidacloprid hydroxylation activity and degraded 58.9% acetamiprid within 8 days of incubation and generated the (E)-*N*-[(6-chloro-3-pyridyl)-methyl]-*N*-cyano-acetamidine (IM 2-1) intermediate by the demethylation process (Chen et al., 2008). With the production of *N*-methyl-(6-chloro-3-pyridyl) methylamine, *Pigmentiphaga* sp. AAP-1 degraded 100 mg⋅L^–1^ acetamiprid within 2.5 h (Wang et al., 2013a). Yang et al. (2013) reported that 0.22 mM acetamiprid was completely degraded by *Pigmentiphaga* sp. D-2 after 72 h. *Pseudoxanthomonas* sp. AAP-7 was reported to hydrolyze or demethylate acetamiprid to 1-(6-chloropyridin-3yl)-*N*-methylmethanamine as an intermediate (Wang et al., 2013b). *Rhodococcus* sp. BCH2 was reported to degrade acetamiprid into the intermediate *N*-methyl (6-chloro-3-pyridyl) methylamine and 6-CNA with glucose and ammonium chloride (Phugare and Jadhav, 2013).

In addition to bacterial strains, fungi also possess better acetamiprid bioremediation potential. Fungal hyphae secrete extracellular enzymes that can penetrate deeper into the soil matrix with a larger surface area than bacterial isolates (Sun et al., 2017; Shi et al., 2018; Guo et al., 2019). Isolating from rotten wood, a white-rot fungus *Phanerochaete sordida* YK-624 degraded acetamiprid to IM 2-1 over 15 days (Wang et al., 2012). The yeast *Rhodotorula mucilaginosa* strain IM-2 degraded acetamiprid as well as thiacloprid in both cultures and soil (Dai et al., 2010).

### Thiacloprid

Thiacloprid, [3-((6-chloro-3-pyridine) methyl)-1,3-thiazolin-2-subunit] melamine, is a chlorinated nicotinoid insecticide especially used to manage stinging and chewing insects. The mechanism of this pesticide is different from traditional pesticides as it affects the articulation of the posterior nerve membrane in insects. Nicotinic acetylcholine receptors interfere with normal conduction to block nerve channels in the insect nervous system. This results in a substantial accumulation of acetylcholine that severely excites the insect’s body to cause body spasms and paralysis leading to death. It quickly produces strong shock, stomach poison, and internal absorption for a longer duration.

Under the soil, laboratory, or field conditions, the half-life of thiameacloprid ranged from 5 to 27 days, indicating that its transformation was strongly affected by soil microbial activity (Liu et al., 2011). Two bacterial strains, *Variovorax boronicumulans* CGMCC 4969 and *Ensifer meliloti* CGMCC 7333, and a yeast, *Rhodotorula mucilaginosa* IM-2, hydrolyzed thiacloprid to thiacloprid amide with half-lives of 14.3 days, 1.8 days, and 20.9 h, respectively (Zhao et al., 2009; Liu et al., 2011; Zhang et al., 2012; Ge et al., 2014).

*Maltophilia oligotrophomonas* CGMCC 1.1788 degraded 90.5% of 0.63 mmol⋅L^–1^ thiacloprid into 4-hydroxyl mercaptoimidacloprid through hydroxylation, within 30 h at a half-life of 9 h. However, at higher concentrations, the rate of transformation was very slow and after 5 days of culture, only 24.2% thiacloprid was converted (Zhao et al., 2009). *Microvirga flocculans* CGMCC 1.16731, an N_2_-fixing bacterium, is not only a biofertilizer agent but also effectively degrades thiacloprid and acetamiprid through NHase mediated transformation (Zhao et al., 2019). This bacterium belongs to the genus *Proteus*, a recently discovered root nodule bacterium, whose environmental functions are still poorly understood (Tomasz et al., 2018).

### Thiamethoxam

Thiamethoxam,3-(2-chloro-1,3-thiazol-5-yl-methyl)-5-methyl-1,3,5-oxadiazinan-4-ylidene (nitro) amine, is another neonicotinoid insecticide that shares similar mechanisms with imidacloprid and has no interactive resistance to imidacloprid, idinidine, or alkenididine. The metabolism of microbial degradation of thiamethoxam in soil and liquid culture has only been reported through a few species, including *Ensifer adhaerens* TMX-23, a nitrogen-fixing and plant-growth-promoting rhizobacterium (Zhou et al., 2013), *Pseudomonas* sp. (Pandey et al., 2009), *Bacillus amyloliquefaciens* IN937a, *Bacillus pumilus* SE34, *Sphingomonas* sp. TY, and nicotine-degrading bacteria *Acinetobacter* sp. TW (Wang et al., 2011; Rana et al., 2015). *Aerophilus* sp. IMBL 4.1 and *Pseudomonas putida* IMBL 5.2 showed significantly higher thiamethoxam degradation potential and were able to grow under oscillating conditions of temperature and pH (Rana et al., 2015). *Ensifer adhaerens* TMX-23 possesses bioaugmentation potential and promoted the growth of field crops in soil contaminated with thiamethoxam (Zhou et al., 2013).

### Clothianidin

Clothianidin, (E)-1-(2-chloro-1,3-thiazol-5-yl-methyl)-3-methyl2-nitroguanidine, is a kind of new nicotinic insecticide with high efficiency, safety, and selectivity. Its effect was similar to that of the nicotinic acetylcholine receptor and caused shock, gastric toxicity, and internal absorption. Clothianidin is mainly used in rice, vegetables, fruit trees, and other crops to control hemipteran, coleopteran, dipteran, and some lepidopteron pests. The microbial degradation of clothianidin under aerobic and anaerobic conditions was studied. The rate constant (*k*) and half-life (DT_50_) of aerobic and anaerobic microorganisms were determined, and enrichment experiments were carried out under different nutritional conditions.

Microbial growth was assessed at different pesticide concentrations and temperatures (Mulligan et al., 2016). Mori et al. (2017) reported the microbial biodegradation of clothianidin. They found that 37% of the clothianidin was degraded by the white-rot fungus *Phanerochaete sordida* during 20 days of cultivation. This revealed that the degradation process of clothianidin in *sordida* is similar to that of mice but the microbial metabolism of clothianidin has not been described well. *Pseudomonas stutzeri* smk aerobically degraded 62% of clothianidin within 14 days at 30°C, which is faster than reported earlier (Parte and Kharat, 2019).

### Nitenpyram

Nitenpyram (NIT), [(E)-*N*-(6-chloro-3-pyridylmethyl)-*N*-ethyl-N0-methyl-2-nitrovinylidenediamine], is a new nicotinoid insecticide product developed in Japan after imidacloprid and acetamiprid. It shares excellent absorbability, osmotic action, and wide insecticidal spectrum properties. It is less harmful and safer than imidacloprid; thus, it can be frequently used to prevent and control pests of stinging mouthparts, such as whiteflies, aphids, pear psyllids, leafhoppers, thrips, etc (Wang J. et al., 2019). Until now, only one fungal strain belonging to white-rot fungus *Phanerochaete sordida* YK-624 was reported to degrade nitenpyramm. Under ligninolytic conditions, *P. sordida* YK-624 completely degraded nitenpyram while there was only a 20% decrease under non-ligninolytic conditions. However, the microbial degradation mechanisms of nitenpyram are not yet explored (Wang J. et al., 2019).

### Dinotefuran

Dinotefuran (DIN), *N*-methyl-N0-nitro-N00-[(tetrahydro-3-furanyl) methyl] guanidine, is another nicotinoid insecticide, with high insecticidal activity at very low doses. Like nitenpyram, it is also very safe for mammals and not well reported for microbial degradation. However, a similar white-rot fungus, *P. sordida* YK-624, was found capable of degrading 31% of dinotefuran in 20 days under ligninolytic conditions. *P. sordida* YK-624 did not degrade dinotefuran without ligninolytic conditions. The microbial degradation mechanisms of dinotefuran are not yet understood (Wang J. et al., 2019).

## Metabolic Pathways of Neonicotinoid Degradation in Microorganisms

### Imidacloprid

**FIGURE 1:**
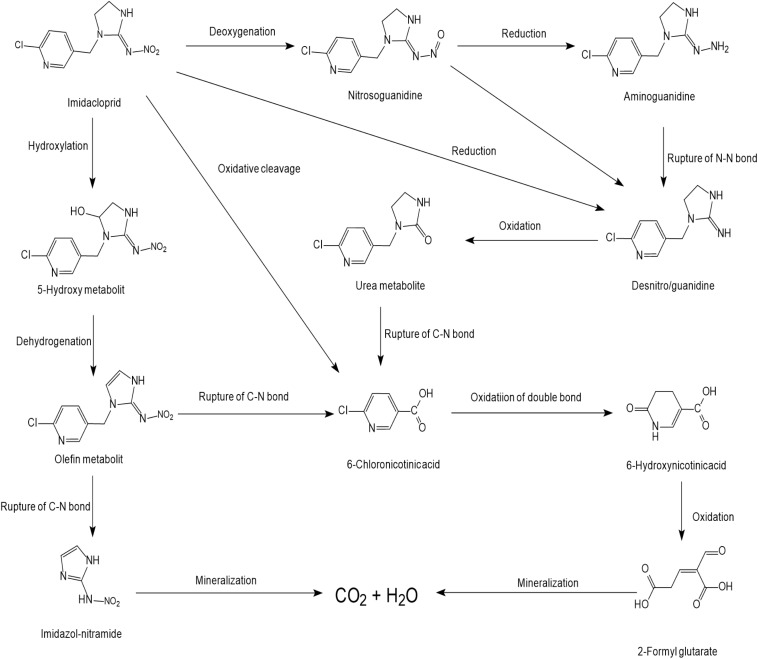
Microbial degradation pathways of imidacloprid (based on Akoijam and Singh, 2014; Sharma et al., 2014; Sabourmoghaddam et al., 2015).

Many imidacloprid-degrading microorganisms and their reported metabolic pathways are presented in Figure 1. Oxidation and nitro-reduction are two major microbial biodegradation pathways of imidacloprid (Akoijam and Singh, 2015; Lu et al., 2016; Fusetto et al., 2017). The products produced by light or water degradation and plant metabolisms include imidacloprid urea, 6-chloronicotinic aldehyde, 6-chloro-*N-*methylnicotinacidamide, 6-chloronicotinic acid, etc. Soil microbial studies indicate that these products may be metabolites of imidacloprid biodegradation (Sabourmoghaddam et al., 2015).

Among the residues of imidacloprid metabolic products in soil, urea is mostly found followed by 6-CNA, 5-hydroxy, olefine, nitrosimine, and nitroguanidine. These residues were not detectable after 60 to 90 days in the second use of imidacloprid (Akoijam and Singh, 2014). Nitro-reduction is the most common and effective approach for imidacloprid bioremediation (Pandey et al., 2009; Sharma et al., 2014).

Initially, two oxygen atoms are removed from imidacloprid to form nitrosoguanidine and aminoguanidine, respectively. The cleavage of the N–N bond produces matter that can form a desnitro/guanidine intermediate with ten times the toxicity to imidacloprid. Intermediate further oxidizes to non-toxic metabolite urea (Pandey et al., 2009; Phugare et al., 2013). In different bacterial systems, matter transforms into 6-chloronicotinic acid due to the cleavage of the C–N bond (Sharma et al., 2014). 6-chloronicotinic acid is easy to decompose organic matter, which generates CO_2_ and H_2_O after oxidation.

The oxidation pathway is less responsive in the microbial systems and generates comparatively fewer degradation products. Imidacloprid forms 5-hydroxy metabolites and olefin metabolites via ethylene hydroxylation and the dehydrogenation pathway (Dai et al., 2006; Ma et al., 2014). The carbon atom of the tertiary amine is connected with the 6-chloro-3-pyridinylmethyl moiety of imidacloprid, which is an important active site of hydroxylation (Dai et al., 2010). After the cleavage of the C–N bond, the olefin metabolite is converted to imidazole-nitramide and 6-chloronicotinic acid. Similarly, this mechanism of imidacloprid degradation also exists in animals and several plant species (Ford and Casida, 2006; Thurman et al., 2013). In addition to this, the *Cyp6g1* gene found in drosophila plays a crucial part in the degradation of imidacloprid in animals to control and promote the production of metabolites in the oxidation pathway (Fusetto et al., 2017).

### Acetamiprid

**FIGURE 2:**
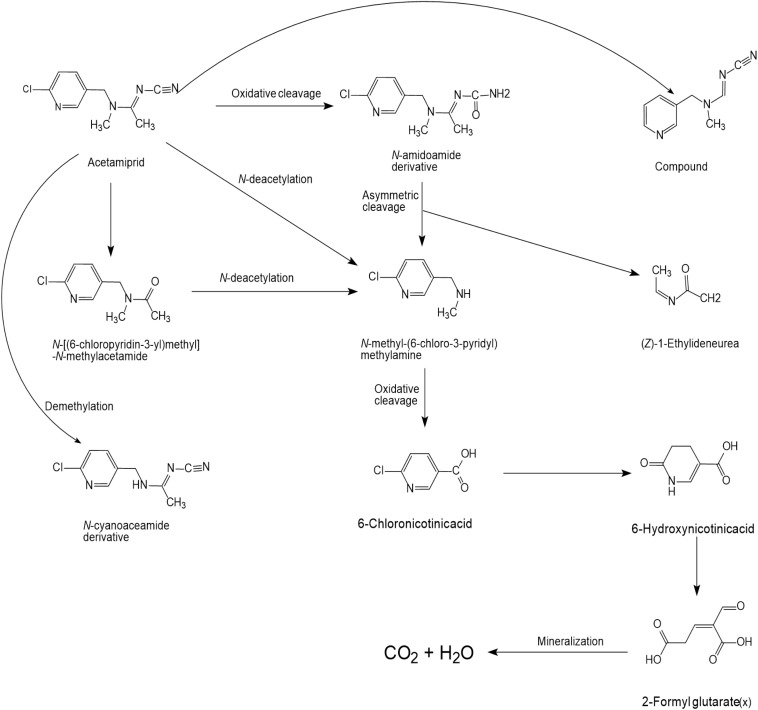
Microbial degradation pathways of acetamiprid (based on Phugare and Jadhav, 2013; Sun S. et al., 2018).

**FIGURE 3:**
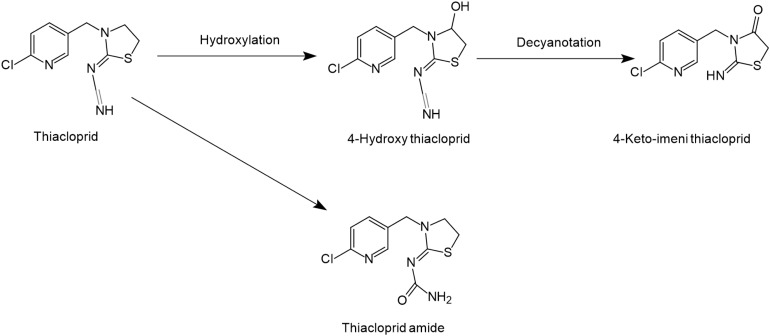
Microbial degradation pathways of thiacloprid (based on Zhao et al., 2009; Thurman et al., 2013).

Several articles have reported the microbial degradation of acetamiprid, as shown in Figure 2 (Tang et al., 2012; Wang et al., 2013a, b; Yang et al., 2013; Shi et al., 2018). Microbial growth mainly depends on the availability of nutrients and their metabolic activity is regulated by the nutrient state of the medium (Phugare and Jadhav, 2013). The insecticidal selectivity of acetaminidine depends on the substituent = NCN and this functional group also initiates the degradation. Generally, the C≡N of acetamiprid is oxidized and fractured to generate *N*-amidoamide derivative. Due to the asymmetric cleavage, product is degraded to *N-*methyl-(6-chloro-3-pyridyl) methylamine and (*Z*)-1-ethylideneurea. The intermediate product quickly generates 6-chloronicotinic acid, which is finally mineralized to H_2_O and CO_2_ (Figure 3; Phugare and Jadhav, 2013; Sun S. et al., 2018).

Tang et al. (2012) found that *Stenotrophomonas* sp. THZ-XP transformed acetamiprid into *N*-methyl-(6-chloro-3-pyridyl) methylamine through the intermediate product. Wang et al. (2013a, b) found that acetamiprid can be directly converted into a product without generating any intermediate products. Interestingly, another pathway was reported in *Ochrobactrum* sp. D-12 to directly dechlorinate and demethylate acetamiprid into the product (Yang et al., 2013).

*Stenotrophomonas maltophilia* CGMCC 1.1788 transformed acetamiprid to a polar metabolite by reducing its one carbon and two hydrogen atoms. Demethylation of acetamiprid generated a *N*-cyanoaceamide derivative that has a lower insecticidal activity (Chen et al., 2008). Interestingly, a common inhibitor of cytochrome P450, known as piperonyl butoxide, restrained the *N*-demethylation of acetamiprid (Chen et al., 2008). However, the microbial system could not transform the *N*-cyanoaceamide derivative into other products and was only found in animal and plant systems (Brunet et al., 2005).

Acetamiprid metabolism in plants, animals, and soil has been clearly understood along with some examples of microbial degradation. Moreover, ammonase activity, found in many microorganisms, plays an important role in the industrial synthesis of amides. However, the catalytic efficiency and biochemical and structural characteristics of NHase enzymes related to the biodegradation of nitrogenous organic pollutants (especially nitrogenous pesticides) have not been thoroughly studied (Guo et al., 2019). The enzymatic mechanism by which potentially useful environmental microorganisms degrade acetamiprid has not been clearly described (Zhou et al., 2014).

### Thiacloprid

The structure of thiacloprid is similar to acetamiprid and therefore very limited literature is available regarding its microbial biodegradation pathways (Figure 3). Moreover, the degradation pathway of acetamiprid can be used as a reference in degradation studies of thiacloprid. Microbial systems release the cyano group of thiacloprid and oxidize hydroxyl group to the carbonyl group to generate 4-hydroxy thiacloprid. 4-hydroxy thiacloprid is rapidly converted into 4-keto-imeni thiacloprid by the decyanotation process. In these two steps of detoxification, thiacloprid loses the cyano and hydroxyl groups (Zhao et al., 2009). Ge et al. (2014) reported that thiacloprid was converted to thiacloprid amide via oxidative cleavage. In addition, a few studies have also revealed that plants and animals can metabolize thiacloprid but subsequent degradation products have not been reported (Thurman et al., 2013).

### Thiamethoxam

**FIGURE 4:**
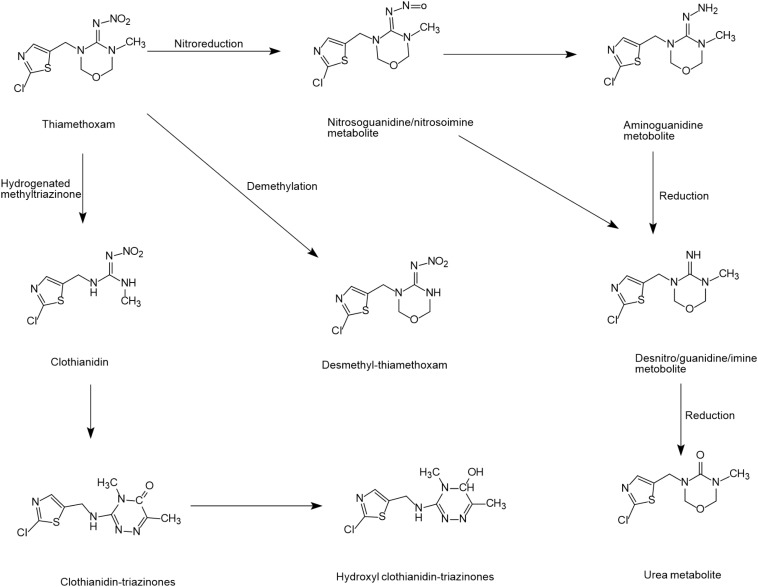
Microbial degradation pathways of thiamethoxam (based on Pandey et al., 2009; Zhou et al., 2014).

Thiamethoxam degradation has been reported in many plants and animals but only a few biodegradation studies have been conducted in microbial systems (Nauen et al., 2003; Ford and Casida, 2008). Microorganisms can degrade thiamethoxam via the nitro reduction metabolic pathway to form metabolites, such as nitrosoguanidine/nitrosamine, amino-guanidine, desnitro/guanidine/imine and urea (Pandey et al., 2009; Zhou et al., 2013). Zhou et al. (2014) reported a biodegradation mechanism by *Ensifer adhaerens* TMX-23 where the cleavage of oxadiazine and hydrogenated methyltriazinone finally converted thiamethoxam into clothianidin-triazinones. Zhou et al. (2014) also reported a biodegradation pathway similar to acetamiprid where thiamethoxam was converted to desmethyl-thiamethoxam through the demethylation pathway. However, the final degradation products were not found in this pathway (Figure 4).

### Clothianidin

**FIGURE 5:**
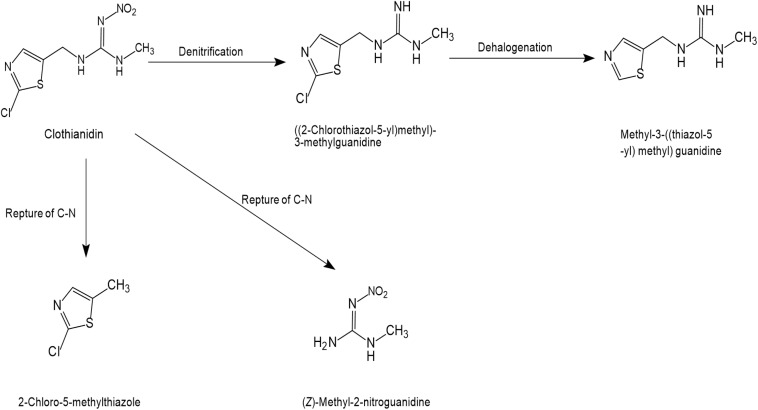
Microbial degradation pathways of clothianidin (based on Parte and Kharat, 2019).

The microbial degradation pathway for clothianidin has only been reported in a few articles, as shown in Figure 5. The cleavage of the C-N bonds between thiazolyl methyl and the guanidine moieties was found to release carbon for the conversion of clothianidin to 2 chloro-5-methyl thiazole and methyl nitroguanidine (Parte and Kharat, 2019). Microbial systems gradually degrade clothianidin to ((2-chlorothiazol-5-yl)methyl)-3-methylguanidine and methyl-3-((thiazol-5-yl) methyl) guanidine through denitrification and dehalogenation. Similar to thiamethoxam and thiacloprid, subsequent degradation products of clothianidin were also not found.

## Molecular Biology of Neonicotinoid Degradation

Pesticides are frequently used in crops to protect them from harmful insects and to increase their productivity and yields. However, the excessive use of pesticides can reduce the activity of soil enzymes, which is a key indicator of soil health. Enzymes plays an important role in the biodegradation of natural and man-made organic compounds in soil and are often used to indicate changes in the soil environments under the action of pesticides and fertilizers. Enzyme activity is closely correlated to microbial activity. To evaluate the effects of pesticides, molecular techniques were used to research the changes in microbial community structure and function (Parween et al., 2016).

The use of molecular tools to identify related genes/enzymes and ineffective bacteria/fungi is of great significance for large-scale and effective bioremediation in pesticide contamination sites. By exploring the molecular basis of biodegradation of pesticides by soil microorganisms, the roles of degrading genes and the application of recombinant DNA technology were reported. Molecular biology techniques provide a more comprehensive explanation of *in situ* microbial communities than standard microbiological methods. At present, the degradation technologies mainly include gel electrophoresis (DGGE), restriction fragment length polymorphism (RFLP), dot blot, Southern blot, PCR amplification, the preparation of metagenomic libraries, the subsequent analysis of bacterial rRNA genes, microarrays, and omics technologies (Parween et al., 2016).

Advances in genomics accelerated the investigation of the novel neonicotinoid degrading gene families. During degradation, the main target for neonicotinoids is the α and β helix proteins (Chen et al., 2017). Many previous researchers explored the details regarding the molecular sites for the neonicotinoids (Matsuda et al., 2020). The biodegradation ability of microorganisms is the key factor in degrading toxic pollutants from the environment. The biodegradation potential of an organism is decided by the genetic content inside individual cells (Feng et al., 2020b). Deoxyribose nucleic acid (DNA) codes the information in the form of mRNA that is further converted to specific degradative enzymes.

During microbial degradation, neonicotinoid degrading enzymes can be up-regulated or down-regulated. To date, the complete degradation of neonicotinoids has not been emphasized. The *Hymenobacter latericoloratus* CGMCC 16346 has been characterized for the degradation of the imidacloprid. This bacterial strain can degrade the imidacloprid through hydroxylation. The whole genome of this strain has been sequenced and compared with other imidacloprid degrading microbial strains. Both chromosome and plasmid genes were shown to participate in the degradation of imidacloprid (Guo et al., 2020).

In insects, detoxifying enzymes, such as esterase, glutathione S-transferase, and P450s, can catabolize insecticides and toxins. These enzymes, involving point mutations in the target gene to the insecticide, could increase the copy number, mRNA levels, and coding sequence diversity of the response gene (Li et al., 2007). The cytochrome P450 is a large, well-defined family of monooxygenases. Their potential has long been recognized in many industrial processes, particularly because of their ability to utilize molecular oxygen for the oxidation or hydroxylation of substrates (Scott et al., 2008). The P450 enzyme carries out various functions, including biosynthesis and metabolism of alien organisms. The insect genome has been found to contain from 46 to more than 150 P450 genes, and each can translate a different P450 enzyme (Table 4).

**TABLE 4:** Enzymes reported for the degradation of neonicotinoid compounds.

Enzymes	Source	Neonicotenoid compound	Specific statement	References
CYP6ER1	*Nilaparvata lugens*	Imidacloprid, Thiamethoxa, dinotefuran	Over-expressed in thiamethoxam-resistant and dinotefuran-resistant strains	Pang et al., 2016
CYP6G1	*Drosophila melanogaster*	Imidacloprid	An enzyme that produces toxic but easily excreted metabolites	Fusetto et al., 2017
CYP6CM1	*Bemisia tabaci*	Imidacloprid	*Bemisia tabaci* resistant to imidacprid lacks resistance to dinotefuran	Hamada et al., 2019
CYP6Y3	*Myzus persicae*	Neonicotinoids	At least one copy of CYP6CY3 with a different promoter sequence may be relevant and requires further study	(Wondji et al., 2008)
CYP353D1v2	*Laodelphax striatellus*	Imidacloprid	Metabolize imidacloprid to 5-hydroxy-imidacloprid	Elzaki et al., 2017
CYP6CY14	*Aphis gossypii*	Thiamethoxam	RNA interference targeting CYP6CY14 increased the sensitivity of resistant aphid to thiamethoxam	Wu et al., 2018

At present, the overexpression of one or more P450 enzymes appears to be the main pathway of pest resistance against new toxoid (Nelson, 2009). In insects, resistance genes were noted to be responsible for neonicotinoid degradation, and similar genes carry out the degradation of neonicotinoids in bacteria. Studies regarding neonicotinoid metabolites and the total enzyme activity have revealed that the development of resistance against this insecticide group is due to P450 monooxygenase rather than mutations of nAChR (Rauch and Nauen, 2003). Several P450 genes and enzymes of the CYP6 and CYP3 clade such as P450 CYP6ER1, CYP6G1, CYP6Y3, and CYP353D1v2 have been well reported for their role in neonicotinoid resistance.

P450 CYP6ER1 was connected with the resistance of imidacloprid resistance in *Nilaparvata lugens*, where amino acid substitutions in the binding site directly contributed to enhancing the metabolism of imidacloprid (Bao et al., 2016; Pang et al., 2016). The CYP6G1 enzyme was used to characterize and quantify the imidacloprid metabolism. It highlights the importance of undetermined transporters in response to imidacloprid by producing toxic metabolites, which were easily excreted (Fusetto et al., 2017). Different from *Nilaparvata lugens* CYP6ER1, all studied CYP6CM1 variants in *Bemisia tabaci* yielded similar levels of imidacloprid metabolism (Hamada et al., 2019). Puinean et al. (2010) indicated that CYP6Y3 could confer resistance to neonicotinoid in *Myzus persicae* (Puinean et al., 2010). CYP353D1v2 was found to over-express in different strains of imidacloprid-resistant *whoes striatellus* and the RNAi of this gene could significantly suppress the resistance (Elzaki et al., 2017). In the overexpressed P450 gene of the CYP3 clade, successful inhibition of CYP6CY14 transcription by RNAi significantly increased the susceptibility of pesticide-resistant cotton aphids to thiamethoxam (Wu et al., 2018).

## Conclusion and Future Prospects

This review comprehensively summarized the microbial degradation and biochemical mechanisms of neonicotinoids. Neonicotinoid pesticides are commonly used in agricultural sectors due to their effective insecticidal properties. However, the deep-rooted negative environmental effects of neonicotinoids should be given serious attention to remove these residues from polluted environments. The application of efficient neonicotinoid-degrading microorganisms in contaminated environments is considered to be the most promising remediation strategy. The toxicity and microbial degradation pathways of imidacloprid, acetamiprid, thiacloprid, thiamethoxam, and clothianidin in neonicotinoids have been clearly understood; however, there are only a few studies regarding nitenpyram and dinotefuran.

The majority of the neonicotinoid degradation intermediates (especially of imidacloprid) are more toxic than their parent compound. Researchers have explored potent microorganisms from pesticide-contaminated agricultural soils, wastewater, rhizospheric soils, and microbial preservation centers. It is noteworthy that none of the single bacterial isolates has been able to fully mineralize imidacloprid, acetamiprid, thiacloprid, thiamethoxam, or clothianidin. Imidacloprid and acetamiprid can be completely co-degraded into carbon dioxide and water by different microorganisms. The isolation or design of such bacteria is critical for the long-term success of biologically mediated environmental degradation of imidacloprid and other neonicotinoids.

To date, only a few reports have conclusively determined the degradation pathways and their associated enzymes. To treat neonicotinoids systematically, the bioremediation potential of degrading microorganisms and enzymes deserves more research. To understand the degradation mechanisms in a contaminated environment, it is very important to study functional genes and enzymes. An immense amount of neonicotinoid-degrading microorganisms have been affirmed; however, few studies have been conducted on their functional genes and enzymes. Therefore, detailed basic work should be carried out before large-scale applications of neonicotinoid-degrading microorganisms for bioremediation. The results of previous studies indicated that high-output sequencing methods may be helpful for the complete annotations of the genes and metabolites produced during microbial degradation.

## Author Contributions

SC conceived of the presented idea. SP contributed to the writing and prepared the figures and tables. ZL, WZ, SM, PB, and SC participated in revising the manuscript. All authors approved it for publication.

## Conflict of Interest

The authors declare that the research was conducted in the absence of any commercial or financial relationships that could be construed as a potential conflict of interest.
